# Characteristics of individuals presenting to treatment for primary alcohol problems versus other drug problems in the Australian patient pathways study

**DOI:** 10.1186/s12888-016-0956-9

**Published:** 2016-07-19

**Authors:** Dan I. Lubman, Joshua B. B. Garfield, Victoria Manning, Lynda Berends, David Best, Janette M. Mugavin, Tina Lam, Penny Buykx, Andrew Larner, Belinda Lloyd, Robin Room, Steve Allsop

**Affiliations:** Turning Point and Monash University, 54-62 Gertrude Street, Fitzroy, VIC 3065 Australia; Centre for Health and Social Research, Australian Catholic University, Level 5, 215 Spring Street, Melbourne, VIC 3000 Australia; Room HC.2.14, Heart of the Campus Building, Collegiate Crescent, Collegiate Campus, Sheffield, S10 2BQ UK; Centre for Alcohol Policy Research, La Trobe University, 215 Franklin St., Melbourne, 3000 VIC Australia; National Drug Research Institute, 10 Selby Street, Shenton Park, WA 6008 Australia; University of Sheffield School of Health and Related Research (ScHARR), Regent Court, 30 Regent Street, Sheffield, S1 4DA UK

**Keywords:** Substance use disorder, Alcohol, Drug, Substance use treatment, Socioeconomic disadvantage, Quality of life, Service use

## Abstract

**Background:**

People seeking treatment for substance use disorders often have additional health and social issues. Although individuals presenting with alcohol as the primary drug of concern (PDOC) account for nearly half of all treatment episodes to the Australian alcohol and other drug (AOD) service system, previous treatment cohort studies have focused only on the profile of Australian heroin or methamphetamine users. While studies overseas indicate that clients seeking treatment primarily for their drinking are less likely to experience social and economic marginalisation than those seeking treatment primarily for illicit or pharmaceutical drug use, very little research has directly compared individuals presenting with alcohol as the PDOC to those primarily presenting with other drugs as their PDOC.

**Methods:**

Seven hundred and ninety-six participants were recruited at entry to specialist AOD treatment in Victoria and Western Australia, and completed measures of demographic and social factors, substance use, quality of life, service use, and criminal justice involvement. We compared those with alcohol as their PDOC to those with other drugs as their PDOC using Pearson chi-square and Mann–Whitney U tests.

**Results:**

Rates of social disadvantage, poor quality of life, high severity of substance dependence, and past-year AOD, mental health, acute health, and social service use were high in all groups. However, participants with alcohol as the PDOC were older; more likely to have an educational qualification; less likely to report criminal justice involvement, housing/homelessness service use, tobacco smoking, or problems with multiple substances; and reported better environmental quality of life; but were more likely to have used ambulance services, than those with other drugs as their PDOC.

**Conclusions:**

While those seeking treatment primarily for alcohol problems appear less likely to suffer some forms of social and economic disadvantage or to use multiple substances than those with a primary drug problem, they experience similarly high levels of substance dependence severity and mental health and AOD service use. These findings reinforce the need for AOD services to integrate or coordinate care with programs that address the many complexities clients frequently present with, while also acknowledging differences between those seeking treatment for alcohol versus other drug problems.

## Background

The significant health harms and social costs associated with alcohol and other drug (AOD) use disorders are well-documented [1–4]. However, recent studies from Australia indicate that less than a quarter of people with an alcohol use disorder in the past year seek any type of mental health treatment [5], while rates of help-seeking among young people with substance use disorders are even lower [6]. The stigmatisation involved in seeking help for AOD problems [7] contributes to low levels of help-seeking for these disorders, and leads people to delay help-seeking until their substance use problems begin to affect multiple domains of their lives. Thus, when people do seek treatment, they often present with additional health and social issues, including mental health disorders, unemployment, unstable housing and criminal justice involvement [8–13], highlighting the need for coordinated inter-agency responses.

A small number of studies suggest that clients seeking treatment primarily for their drinking are less likely to experience social and economic marginalisation than those seeking treatment primarily for illicit or pharmaceutical drug use. For example, a recent large study from Singapore (*n* = 563) found higher rates of marriage and employment and better quality of life among individuals presenting for alcohol problems compared to those with other drug problems [14]. Similar findings were reported by Stenius and colleagues [15] in their analysis combining data from AOD treatment clients from Contra Costa County, California and Stockholm, Sweden. The authors categorised participants as “marginalised” (no stable housing; no employment, study, or household duties; and no social networks other than those mainly composed of people with substance use problems), “integrated” (having both stable housing and either employment, study, or household duties), and “intermediate” (having either stable housing or either employment, household duties, or studying, but not both). In both study settings (Sweden and California), marginalised and intermediate participants were more likely than integrated participants to be dependent on drugs other than alcohol, while in Sweden, the integrated participants were more likely than marginalised participants to be heavy/dependent drinkers. While these data suggest alcohol problems are associated with less social marginalisation than other drug problems, an analysis of mortality rates among AOD treatment service clients following treatment in the Australian state of Victoria found that those with alcohol as the primary drug of concern (PDOC) had higher post-treatment mortality rates than clients with any other PDOC, even after controlling for their older age and other demographic differences [16]. This suggests that those with alcohol as their PDOC may experience particularly severe health problems, relative to those with other drug problems.

Publicly-available AOD treatment statistics from England and Australia also suggest demographic differences between clients presenting primarily with alcohol problems and those with other drug problems. In England in 2014–15, clients seeking treatment for alcohol only were, on average, older than the English median age, while clients seeking treatment for illicit drug use, or for a combination of alcohol and illicit drug use, were, on average, below the English median age. Men comprised the majority in all groups, although were a lower proportion among those seeking treatment for alcohol only (62 % vs. 73–75 % in illicit drug-using groups). Similar to the findings of less marginalisation in Sweden and Singapore, alcohol-only clients in England were less likely to report housing problems compared with clients with other drug problems [17]. Consistent with these findings, data on AOD treatment episodes in 2012–13 in Australia [18] highlight alcohol as more commonly being the PDOC among older age groups than among younger treatment entrants and, relative to clients for whom cannabis, amphetamines, or heroin was the PDOC, those with alcohol as the PDOC included a larger proportion of female clients. In addition, those with alcohol as the PDOC were more likely to be Aboriginal or Torres Strait Islander and less likely to report secondary drugs of concern. Alcohol was also more likely to be the PDOC in remote areas than in major cities.

In the Australian publicly-funded AOD treatment system, substance use is assessed and treated within a single specialist service system, with clients usually treated by the same workforce within the same services regardless of their PDOC. Treatment may include outpatient counselling, withdrawal management (in a residential detoxification facility or home-based), rehabilitation (usually residential), information/education interventions, or case management to support other health and welfare needs. Opiate substitution pharmacotherapy is offered by AOD specialist treatment services in some Australian states, but is primarily accessed through primary health care services (i.e. general practitioners). The most common PDOCs among clients accessing publicly funded AOD specialist treatment services in Australia are alcohol and cannabis (equating to 41 and 24 % of treatment episodes, respectively in 2012–2013), followed by amphetamines (14 %) and opioids (13 %) [18]. In contrast, the two largest Australian treatment outcome studies conducted to date primarily focussed on clients with problematic opioid [12] or meth/amphetamine use [19], and the characteristics of those presenting for treatment for alcohol or cannabis use within the Australian system has received little research attention. It remains unclear whether differences in the “marginalisation profile” found between alcohol-dependent and other drug-dependent clients in Sweden, Singapore and the US generalise to Australia, and to what degree health and other factors (e.g., poor health, criminal involvement) further complicate presentations to the AOD system. Moreover, while international studies have shown high rates of acute health service use [20, 21], broader health and welfare service system use by this population has not been examined in detail in Australian treatment cohort studies to date.

In the Patient Pathways study, we aimed to address these issues by interviewing clients from AOD services in two Australian states at treatment entry to assess their pathways into treatment, service use, and the nature and severity of their problems. In this paper, we describe characteristics of participants at the baseline interview, with a specific focus on differences between those reporting alcohol, as opposed to other drugs, as their PDOC. Based on treatment cohort studies internationally, and recent Australian treatment data, we expected those reporting alcohol as their PDOC would be older, less socially marginalised (i.e. more likely to be employed, to have educational qualifications, to be in a relationship) and to therefore report a higher social and environmental quality of life, as well as being less likely to report problems with additional substances, than those reporting other drugs as PDOCs. Given the different legal status of alcohol compared with most other commonly-used drugs, we also predicted that those with alcohol as the PDOC would report lower rates of legal problems. We expected these differences to also be reflected in lower rates of use of certain types of social services, including housing, employment, and legal aid services, among those with alcohol as the PDOC. However, given previous findings of higher mortality rates in those with alcohol as the PDOC, we also expected they would report a higher rate of health service use.

## Method

### Participants and procedure

AOD treatment services in Victoria and Western Australia (WA) were purposively selected as recruitment sites to represent major treatment types. In selecting agencies, both metropolitan and regional locations were included, with a preference for agencies with a substantial client load and offering two or more treatment types. Of 21 organisations approached for participation, all but one agreed to be involved.

Baseline interviews were conducted between January 2012 and January 2013. Interviews were conducted face-to-face (except in 11 cases where distance necessitated a telephone interview) by trained researchers. Clients were eligible for the study if they were at least 18 years of age; had been assessed for, or commenced, their primary index treatment (PIT) episode in the past month; and had not engaged in the same treatment in the three weeks prior to commencing the current treatment episode. In total, 1054 clients were referred to the study for screening, of whom 796 (75.5 %) met inclusion criteria, provided written informed consent, and completed the baseline interview. Of the remainder, 81 were found to be ineligible, 26 declined to participate, 1 was deceased, and 150 could not be contacted. Of the final sample of 796 people, there were 214 whose PIT was outpatient treatment (assessment *n* = 29, outpatient counselling *n* = 170, pharmacotherapy *n* = 15); 352 whose PIT was withdrawal management (inpatient detoxification *n* = 346, home-based detoxification *n* = 6); and 230 whose PIT was longer-term residential rehabilitation or engagement in a therapeutic community. Participants were reimbursed $25 for the baseline interview, which took an average of one hour. Ethical approval for the study was obtained from Research Ethics Committees at Eastern Health, Monash University, and Curtin University.

### Measures

Demographic and social variables included age, sex, country of birth, Aboriginal or Torres Strait Islander status, employment, income support payment received, housing and legal status. Participants were asked what primary drug of concern (PDOC) brought them to treatment and whether they had any secondary drugs of concern (SDOCs). Dependence on the PDOC was measured using the Severity of Dependence Scale (SDS), which provides a continuous measure that can differentiate severity of dependence, and has been validated in samples of substance users in Australia and internationally [22]. Quality of life (QOL) was measured using the World Health Organisation Quality of Life brief scale (WHOQOL-BREF), which has four domains: psychological, physical, social, and environmental [23].

Participants were asked whether they had accessed AOD (counselling, withdrawal, rehabilitation, and other), health (general practitioner (GP), ambulance, emergency department, hospital inpatient admission, or outpatient mental health), and community (employment, housing/homelessness, legal aid, financial counselling, family/relationship counselling, and other) services in the past year. Using an adapted version of the Lifetime Drug Use History [24], participants were asked to record number of attendances/visits for each service type accessed in the past 12 months. Criminal justice involvement was also recorded in this way.

### Statistical analyses

We conducted analyses using SPSS version 22. Participants were grouped according to their PDOC. Participants who nominated tobacco as their PDOC (*n* = 8) were categorised according to their second DOC, because the specialist AOD treatment system does not typically treat tobacco as the main PDOC, and we therefore assumed that those participants were primarily receiving treatment for their SDOC. Our primary analyses compared participants with alcohol as their PDOC to those with other drugs as their PDOC. Additionally, we conducted pairwise comparisons of those with alcohol as their PDOC to the other 3 main categories of PDOCs: cannabinoids (cannabis and synthetic cannabinoids), opioids (heroin and pharmaceutical opioids), and stimulants (amphetamines, ecstasy, and cocaine). Pearson chi-square tests were used for categorical variables. All continuous variables (age, SDS scores, WHOQOL scores, and number of GP attendances) had non-normal distributions (Kolmogorov-Smirnov ps < .001), so descriptive data for these variables are expressed as medians and inter-quartile ranges, and between-group differences in these variables were analysed with Mann–Whitney U tests. Because large sample sizes, such as ours, may allow minor differences between groups to reach conventional criteria for statistical significance, we instead interpreted between-group differences according to effect size (ES) indices (Cramer’s *V* for chi-square tests; *r* for Mann–Whitney U tests), applying conventional criteria for ES description (0.1 = small; 0.3 = medium; 0.5 = large). We chose to only interpret differences with ES > 0.1 as being substantial, which is a more conservative approach than using the conventional criterion of *p* < .05: all differences in our analyses with ES > 0.1 had *p* < .05, but not all differences with *p* < .05 had ES > 0.1.

Due to our sampling strategy, withdrawal and long-term residential types of treatment were over-represented, and assessment and counselling under-represented, relative to the actual frequency of these treatment types in the specialist AOD treatment system in Victoria and WA. Therefore, weightings based on PIT type were applied to analyses so that results would be more generalisable to the population receiving the treatment types sampled in these states. These were calculated according to the proportions of closed treatment episodes (for those accessing treatment for their own AOD use only) of the treatment types of assessment, counselling, withdrawal, and rehabilitation in 2011–12 for all treatment provided in Victoria and WA reported in the Alcohol and Other Drug Treatment Services National Minimum Data Set [25]. This treatment episode data does not include numbers of episodes with pharmacotherapy as the main treatment type in Victoria or WA, and opioid substitution pharmacotherapy is typically accessed through GPs, rather than the specialist AOD treatment system, in these states, so the 15 participants who reported this PIT type were excluded from weighted analyses (it should be noted, however, that of the remaining participants with opioids as their PDOC, 45.9 % were prescribed opioid substitution pharmacotherapy at the time of their interview). The size of the sample analysed in this report was therefore 781.

## Results

Participants reported the following PDOCs (unweighted ns, weighted/unweighted percentages): alcohol (*n* = 375, 46.0 %/48.1 %); cannabis (*n* = 117, 17.8 %/15.0 %); synthetic cannabinoids (*n* = 2, 0.1 %/0.3 %); heroin (*n* = 76, 11.3 %/9.7 %); painkillers (*n* = 15, 2.3 %/1.9 %); buprenorphine (*n* = 6, 1.0 %/0.8 %); methadone (*n* = 5, 0.9 %/0.6 %); other/unspecified opioids (*n* = 6, 0.7 %/0.8 %); meth/amphetamine (*n* = 155, 17.1 %/19.9 %); cocaine (*n* = 3, 0.4 %/0.4 %); ecstasy (*n* = 1, 0.4 %/0.1 %); benzodiazepines (*n* = 8, 0.5 %/1.0 %); GHB (*n* = 2, 0.1 %/0.3 %); solvent/volatile inhalants (*n* = 1, 0.1 %/0.1 %), and tobacco (*n* = 8, 1.4 %/1.0 %). PDOC was missing for one participant. After classifying the 8 participants who nominated tobacco as their PDOC according to their second DOC (alcohol: *n* = 3; cannabis: *n* = 1; heroin: *n* = 2; meth/amphetamine: *n* = 2), the final categories used for between-group analyses were alcohol (*n* = 378, 46.4 %/48.5 %) and other drugs (*n* = 402, 53.6 %/51.5 %), with the main sub-groupings of other drugs being cannabinoids (*n* = 120, 18.0 %/15.4 %); opioids (*n* = 110, 16.7 %/14.1 %), and stimulants (*n* = 161, 18.2 %/20.6 %).

### Representativeness of the sample

Weighted characteristics of the sample were compared to data on all publicly-funded Australian closed counselling, assessment, withdrawal, and rehabilitation treatment episodes for drug use in 2011–12 [25] on variables that were available to analyse from the latter dataset, to estimate the degree to which our sample was representative of the overall Australian population seeking AOD treatment types included in our sample. As shown in Table 1, our sample appeared to be closely representative in terms of age (except for the youngest age group, due to our exclusion of participants aged under 18) and PDOC, but contained somewhat fewer males and Aboriginal or Torres Strait Islanders than would be expected if we had recruited a random sample from all Australian states and territories.

**Table 1:** Comparison of key sample characteristics to 2011–12 Australian treatment episode data

	Total sample (*N* = 781)	Australian counselling, withdrawal, assessment, and rehabilitation closed treatment episodes for drug use in 2011–12 (*N* = 117,257)
Male (%)	59.6	68.0
Age		
10–19 (%)	1.1	8.7
20–29 (%)	25.8	28.3
30–39 (%)	33.3	30.3
40–49 (%)	24.6	20.8
50–59 (%)	12.4	8.9
60+ (%)	2.8	2.9
Aboriginal or Torres Strait Islander (%)	4.9	12.2
Primary drug of concern (prior to re-assignment of participants with tobacco as PDOC)		
Alcohol (%)	46.0	47.8
Cannabis (%)	17.8	20.1
All opioids (%)	16.2	14.5
Stimulants (amphetamines, cocaine, or ecstasy) (%)	17.9	12.6
Nicotine (%)	1.4	1.0
Benzodiazepines (%)	0.5	1.7

### Demographic characteristics

Demographic, socio-economic, and legal characteristics of the sample are shown in Table 2. The majority of the sample was male. Only those with opioids as their PDOC were substantially more likely to be male than those with alcohol. Those with alcohol as their PDOC were older, both when compared to other PDOCs overall, and compared to each specific drug category, with most differences having ES in the medium-large range. Most participants were Australian born. Only those with stimulants as their PDOC were substantially more likely to be born in Australia than those with alcohol as their PDOC.

**Table 2:** Demographic, socio-economic, and legal indicators

	Total sample	Alcohol	All drugs other than alcohol^a^	Cannabinoids	Opioids	Stimulants
Unweighted sample *N*	781	378	402	120	110	161
Basic demographics						
Male (%)	59.6	56.6	62.3	55.3	72.3*	61.3
Age (Median, IQR)	36.5, 29.3–46.0	41.2, 32.3–49.3	33.8**, 28.0–41.3	32.9*, 25.5–46.1	35.8*, 31.6–41.5	32.7**, 27.8–38.2
Australia born (%)	79.6	77.3	81.6	82.3	72.3	88.7*
Aboriginal/Torres Strait Islander (%)	4.9	4.7	5.1	9.2	1.5	4.3
Socio-economic indicators						
Completed year 12, TAFE, university and/or apprenticeship (%)	49.3	59.7	40.4*	34.3*	48.1*	39.4*
No employment (past 90 days) (%)	64.4	59.5	68.8	71.4*	73.1*	62.4
Current unemployment benefits (%)	39.8	33.1	45.5*	38.6	43.8	53.5*
Current sickness/disability benefits (%)	28.7	28.3	29.2	30.0	37.7	20.4
Any homelessness (past 90 days) (%)	20.8	18.2	23.1	13.1	30.8*	25.2
Criminal justice system involvement						
Current criminal justice issue (%)	28.4	22.4	33.7*	23.6	40.5*	37.9*
Prison (past year) (%)	4.4	3.4	5.3	2.1	8.5*	5.6
Community-based offender program (past year) (%)	11.0	5.4	15.8*	13.6*	23.3*	10.6

*IQR* inter-quartile range

Effect size (ES) of pairwise tests relative to alcohol indicated by: *ES > .1, **ES > .3

^a^Drugs combined includes cannabinoids, opioids, and stimulants, as well as benzodiazepines, GHB, and solvent/volatile inhalants

### Social disadvantage

Less than half of the total sample had completed higher secondary education and/or a post-secondary or trade qualification. In comparison, in 2011, 64 % of Australians aged 25–64 held a vocational or higher education qualification [26]. Approximately two thirds of participants had not engaged in any paid employment in the past 90 days and a large majority were receiving government income support (mainly unemployment or disability payments) at the time of their interview. One fifth had experienced recent homelessness. However, there were several substantial differences between those with alcohol as their PDOC and those with other drugs as their PDOC, with ESs in the small-medium range. Those with alcohol as their PDOC were more likely than the rest of the sample to have completed secondary or post-secondary education. They were also more likely than those with cannabinoids or opioids as their PDOC to have participated in paid employment in the 90 days prior to their interview, and less likely (particularly compared to those with stimulants as their PDOC) to be in receipt of unemployment benefits at the time of the interview. They were also less likely than those with opioids as PDOC to have experienced recent homelessness.

### Criminal justice system involvement

Participants were asked about current criminal justice system involvement including: awaiting charges, trial, sentencing, or court order; summons to appear in court; being on bond; bail; correction orders, treatment orders, and other court orders; parole; suspended sentences; probation; or warrants. Over a quarter of participants had at least one current criminal justice issue. Rates of such problems, however, were substantially lower among those with alcohol as their PDOC than those with other drugs as their PDOC (particularly those with opioids or stimulants as their PDOC). Moreover, those with alcohol as their PDOC were approximately one third as likely as those with other drugs as their PDOC to have been subject to a community-based offender program in the past year, and were less than half as likely to have been imprisoned in the past year than those with opioids as their PDOC, with these differences having small-medium ESs.

### Quality of life

WHOQOL-BREF scores are shown in Table 3. For the sample as a whole, and for both alcohol and drug groups, median scores on every WHOQOL-BREF domain were more than one standard deviation (SD) below the Australian general population norms (using means and SDs reported by Hawthorne, Herrman, and Murphy [27]). Indeed, for psychological QOL, the median for the whole sample, and for those with alcohol as their PDOC, was over 2 SDs below the general population mean. Nevertheless, those with alcohol as their PDOC tended to report substantially better environmental QOL than those with drugs as their PDOC (particularly those with opioids as their PDOC), and also reported substantially better physical QOL than those with opioids as their PDOC, with the ESs for these differences being in the small-medium range.

**Table 3:** Quality of life scores^a^

	Total sample	Alcohol	All drugs other than alcohol^b^	Cannabinoids	Opioids	Stimulants
Unweighted sample *N*	781	378	402	120	110	161
Physical median, IQR	−1.3, −1.9–−0.5	−1.1, −1.9–−0.5	−1.3, −1.9–−0.5	−1.3, −1.9–−0.5	−1.5*, −2.1–−0.7	−0.9, −1.9–−0.3
	(*50.0, 39.3–64.3*)	(*53.4, 39.3–64.3*)	(*50.0, 39.3–64.3*)	(*50.0, 39.3–64.3*)	(*46.4, 35.7–60.7*)	(*57.1, 39.3–67.9*)
Psychological median, IQR	−2.1, −3.0–−0.6	−2.1, −3.0–−0.6	−1.8, −3.0–−0.6	−1.8, −3.0–−0.6	−2.1, −2.7–−0.9	−1.5, −3.0–−0.3
	(*41.7, 29.2–62.5*)	(*41.7, 29.2–62.5*)	(*45.8, 29.2–62.5*)	(*45.8, 29.2–62.5*)	(*41.7, 33.3–58.3*)	(*50.0, 29.2–66.3*)
Social median, IQR	−1.2, −2.6–−0.7	−1.2, −2.6–−0.7	−1.6, −2.6–−0.7	−1.2, −2.1–−0.7	−1.6, −2.6–−0.7	−1.2, −2.3–−0.7
	(*50.0, 25.0–58.3*)	(*50.0, 25.0–58.3*)	(*41.7, 25.0–58.3*)	(*50.0, 33.3–58.3*)	(*41.7, 25.0–58.3*)	(*50.0, 30.5–58.9*)
Environmental median, IQR	−1.2, −2.2–−0.2	−1.2, −1.9–0.0	−1.4*, −2.5–−0.7	−1.2, −2.4–−0.5	−1.9*, −2.9–−1.2	−1.2, −2.4–0.0
	(*59.4, 46.9–71.9)*	(*59.4, 50.0–75.0*)	(*56.2, 43.0–65.6*)	(*59.4, 43.8–68.8*)	(*50.0, 37.8–59.4*)	(*59.4, 43.8–75.0*)

*IQR* inter-quartile range

Effect size (ES) of pairwise tests relative to alcohol indicated by: *ES > .1

^a^To clearly express degree of impairment, relative to the general population, for readers unfamiliar with the WHOQOL scale, WHOQOL scores are expressed as Australian general population z scores (i.e. difference, in Australian general population standard deviations, from Australian general population mean, based on Australian normative data reported by Hawthorne et al. 2006). To allow comparison with international data, scores on the commonly-used 0–100 scale are presented in parentheses, in italics, below z scores

^b^Drugs combined includes cannabinoids, opioids, and stimulants, as well as benzodiazepines, GHB, and solvent/volatile inhalants

### Substance use severity

Following Gossop et al.'s [28] designation of SDS scores over 6 as indicative of severe dependence, Table 4 shows that large majorities of each group were severely dependent on their PDOC (lower quartile cut-off scores all >6). Those with alcohol as their PDOC had substantially higher scores than those with stimulants as their PDOC. Nearly half of the sample had more than one drug of concern, aside from tobacco. However, those with alcohol as their PDOC were substantially less likely than the rest of the sample to have SDOCs – indeed those with opioids as their PDOC were approximately twice as likely as those with alcohol as their PDOC to have SDOCs – and were also less likely to be daily tobacco smokers at the time of the interview. Among those with alcohol as a PDOC who did report SDOCs, the majority reported cannabis as an SDOC, while opioids, amphetamines, and benzodiazepines were each reported by between one quarter and one third of these participants.

**Table 4:** Severity of dependence and secondary drugs of concern (SDOC)

	Total sample	Alcohol	All drugs other than alcohol^a^	Cannabinoids	Opioids	Stimulants
Unweighted sample *N*	781	378	402	120	110	161
SDS score for PDOC: Median, IQR	10, 8–13	10, 8–13	10, 8–12	11, 8–13	11, 9–13	10*, 7–11
Two or more DOCs (other than tobacco) (%)	46.0	34.3	56.0*	51.4*	68.5**	48.6*
Alcohol as an SDOC (%)	23.0	n.a.	23.0	24.3	26.9	17.5
Cannabis as an SDOC (%)	24.1	18.3	31.6*	n.a.	26.9	35.9*
Any opioid as an SDOC (%)	10.3	10.3	10.2	16.3	n.a.	4.2
Any stimulant as an SDOC (%)	16.1	8.3	26.6*	21.4*	31.5*	n.a.
Any benzodiazepine as an SDOC (%)	12.4	11.4	13.3	7.9	26.0*	7.0
Daily^b^ tobacco smoking (%)	71.0	60.6	80.2*	83.8*	77.8*	78.2*

*DOC* drug of concern, *SDOC* secondary drug of concern, *SDS* severity of dependence scale, *IQR* inter-quartile range

Effect size (ES) of pairwise tests relative to alcohol indicated by: *ES > .1, **ES > .3

For analyses of specific substances as SDOC, participants with the substance as PDOC were excluded

^a^Drugs combined includes cannabinoids, opioids, and stimulants, as well as benzodiazepines, GHB, and solvent/volatile inhalants

^b^Smoked tobacco on the majority (i.e. 16–30) of the past 30 days

### Past-year service use

Proportions of participants reporting use of various service types are shown in Table 5. Previous AOD service use was reported by 61.7 % of the whole sample, with outpatient counselling being the most common AOD service type. Those with alcohol as their PDOC used each main AOD service type at similar rates to those with other drugs as their PDOC as a whole, though when compared specifically with those reporting opioids as their PDOC, reported substantially lower rates of counselling and rehabilitation.

**Table 5:** Past year service use

	Total sample	Alcohol	All drugs other than alcohol^a^	Cannabinoids	Opioids	Stimulants
Unweighted sample *N*	781	378	402	120	110	161
AOD services						
AOD Counseling (%)	49.0	46.3	51.3	50.0	57.7*	45.5
Withdrawal (%)	27.2	27.0	27.3	24.1	30.8	27.5
Residential rehabilitation (%)	7.2	6.6	7.9	2.8	14.6*	6.3
Acute medical services						
Ambulance (%)	31.2	37.0	26.1*	22.9*	34.9	21.1*
ED (%)	50.7	54.3	47.5	35.0*	57.7	50.0
Hospital inpatient (%)	27.9	29.2	26.9	29.1	29.2	22.5
GP (%)	92.4	95.0	89.9	86.4*	97.7	86.5*
Median GP visits, IQR	7, 4–13	7, 4–12	7, 4–15	6, 3–12	12*, 5–20	6, 2–12
Other community services						
Outpatient mental health service (%)	45.0	42.7	47.0	48.2	48.8	43.3
Legal aid (%)	30.0	25.1	34.2	27.1	36.9*	38.7*
Employment service (%)	40.9	39.7	42.0	40.0	50.0	35.9
Housing/homelessness service (%)	23.4	18.0	28.0*	26.1	30.2*	26.8

*AOD* alcohol or other drugs, *PIT* primary index treatment, *ED* emergency department, *GP* general practitioner, *IQR* inter-quartile range

Effect size (ES) of pairwise tests relative to alcohol indicated by: *ES > .1

^a^Drugs combined includes cannabinoids, opioids, and stimulants, as well as benzodiazepines, GHB, and solvent/volatile inhalants

Approximately half of the participants had attended an emergency department in the past year, and between a quarter and a third reported being attended to by an ambulance or being admitted to hospital. There were particularly high rates of medical service use among those with alcohol as their PDOC, although rates of acute service use were high in all groups. Relative to those with other drugs as their PDOC (and, particularly, relative to those with cannabinoids or stimulants as PDOC), those with alcohol as their PDOC were substantially more likely to have been attended to by an ambulance. They were also more likely to have visited a GP than those with cannabinoids or stimulants as their PDOC, and were substantially more likely than those with cannabinoids as their PDOC to have attended an emergency department at least once in the past year. However, participants with alcohol as their PDOC reported substantially fewer GP visits than those with opioids as their PDOC. This may reflect accessing of opioid substitution pharmacotherapy by participants in the opioid group, as pharmacotherapy is typically accessed through GPs in the states in which this study was conducted, but may also indicate those with opiate dependence seeking access to pharmaceutical opioids or treatment for complications related to unsafe injecting practices (e.g., cellulitis, blood-borne viruses).

Nearly half the sample accessed outpatient mental health services in the year prior to the interview, and these rates were similar across PDOC groups. Highlighting the frequent legal, housing, and employment problems noted in Table 2, substantial minorities reported accessing legal aid, employment, and housing/homelessness services. Consistent with their lower rates of criminal justice system involvement and recent homelessness, those with alcohol as their PDOC were substantially less likely than those with other drugs as their PDOC to have used housing or homelessness services (particularly relative to those with opioids as their PDOC) and were less likely to have accessed legal aid relative to those with opioids or stimulants as their PDOC. ESs of differences in service use, where they existed, were all within the small-medium range.

## Discussion

Consistent with previous AOD treatment cohort studies [8, 10, 12–15], we found high rates of socio-economic disadvantage, marginalisation, poly-substance use, and health and social service use among people entering publicly-funded AOD treatment in two Australian states. These findings included low rates of educational attainment and employment, a majority of participants reliant on government benefits, and a significant minority suffering homelessness and involvement with the criminal justice system. Participants reported substantially poorer quality of life, relative to Australian norms. A majority had used some additional form of AOD treatment in the previous year, suggesting that chronic or relapsing substance use problems were the norm in this cohort. A majority had also attended acute health services, while nearly half had accessed mental health treatment, and substantial minorities had accessed legal aid and employment services.

These problems were pervasive across the PDOC groupings, suggesting at least some degree of similarity regardless of the PDOC. However, participants with alcohol as their PDOC, who comprised nearly half of the sample, differed in several important ways from participants for whom other drugs were the primary problem. As we expected, those with alcohol as the PDOC were substantially older than other drug groups and, consistent with previous reports from Singapore and Sweden [14, 15], appeared less likely to be socio-economically disadvantaged or marginalised. They were more likely than all other groups to have completed higher secondary or tertiary education or a trade qualification and were less likely to be receiving unemployment benefits (though rates of unemployment benefits were still very high in this group compared to the general population). They were also less likely to have current criminal justice issues or to have served a community-based sentence in the past year. Their lower rate of use of housing/homelessness services in the past year suggests that stable housing was also more common for those with alcohol as their PDOC. Their higher environmental quality of life is likely related to their reduced rates of socio-economic disadvantage, as this scale largely measures living conditions and access to services.

Participants with alcohol as their PDOC were also substantially less likely to report multiple substances of concern. While a majority of those for whom other drugs were the PDOC had multiple DOCs, nearly two thirds of the alcohol group had no additional substance of concern (other than tobacco). Moreover, while a majority of those reporting alcohol as their PDOC were daily tobacco smokers, this was still a substantially lower proportion than among any other group. Consistent with available data on AOD treatment episodes in Australia, these data suggest that poly-substance use is much more common among those seeking treatment for use of drugs other than alcohol, and is less likely to be reported when the PDOC is alcohol.

Despite their apparently lower rates of socio-economic disadvantage, marginalisation, and poly-substance use, participants with alcohol as the PDOC reported similarly poor social, physical, and psychological quality of life ratings as those given by other groups. In particular, psychological quality of life was very low in a majority of participants, both in the alcohol group and in the sample more generally. Taken together with the high rate of mental health service use, which was also similar across groups, these findings are consistent with the high rates of psychiatric comorbidity highlighted within previous AOD treatment studies [5, 8, 10–12]. In addition to mental health problems, high levels of significant physical health problems were also indicated by the high rates of past year acute health service use, with those reporting alcohol as their PDOC substantially more likely to have been attended by an ambulance than the rest of the sample. This higher rate of ambulance attendance, along with Lloyd et al.'s [16] finding of higher post-treatment mortality rates in clients with alcohol as the PDOC, suggests particularly severe health problems within this group. Moreover, despite their lower rates of poly-substance problems, those with alcohol as their PDOC reported similarly high severity of dependence to other groups – indeed substantially higher than the SDS scores reported by those with stimulants as their PDOC – and all groups reported similarly high rates of past year AOD service use.

While the focus of our analyses were on those with alcohol as their PDOC, it is important to note that those with opioids as their PDOC stood out as particularly severe/marginalized on a range of measures. The opioid group reported especially high rates of recent homelessness, criminal justice involvement, secondary drugs of concern (including much higher rates of problems with benzodiazepines than other groups), past-year AOD service use, number of GP visits, and poorer physical and environmental quality of life. These findings are consistent with previous reports of high rates of health and AOD service use, benzodiazepine use, criminal activity, and physical health problems among Australian opioid users [12, 29]. While opioid use has not received the level of public attention that methamphetamine has received in recent years, our results, together with Australian treatment episode data [18], emphasise that opioid use problems are still reported by a substantial proportion of those entering AOD treatment, with a multi-faceted approach likely to be required to address their physical health, housing, and legal problems, in addition to their substance use.

The differential findings by PDOC are likely to reflect two main dimensions of difference between drug classes and those who use them. One is the social position of substance use in Australian society. Use of alcohol is differentiated from non-medical use of other PDOCs not only by its legality, but also by a wide acceptance of use, and of relatively heavy use by young adults. For example, in objecting to an official drinking guideline suggesting an upper limit of four drinks on any occasion, Tony Abbot, then the Minister of Health, spoke in 2008 of a “moral panic”, noting that “what an individual does is his or her responsibility, particularly with something that is legal … We need to know the real enemy, and that is illicit drugs” [30]. In Australian and cognate cultures, adults are expected to cut down their drinking only as they move on in their late 20s to a settled career and forming a family [31], while illicit drug use by young adults is to some extent furtive and much less widely accepted – particularly for heroin and other illicit opiate use. Young people habitually using illicit drugs are thus more likely to encounter informal and formal pressures to enter treatment for drug use, while such pressures concerning habitual heavy use of alcohol normatively occur when they are as much as a decade older. The greater marginalisation of those with illicit drugs as PDOC reflects the selective effects of stigmatisation associated with criminal law and other deterrence [7]. The second dimension of differentiation is that heavy use of alcohol is in many ways more harmful to health than heavy use of most illicit drugs [32, 33]. The multiple health risks of heavy drinking are likely to be a substantial factor in the high rates of GP, ambulance, and emergency department attendance, particularly relative to those with cannabinoids and stimulants as their PDOC.

Nevertheless, most differences between those with alcohol as their PDOC and those with other drugs as their PDOC had ESs that, according to conventional criteria, would be considered small or medium in magnitude. Thus, while it appears that real differences exist at the group level, there is likely to be considerable overlap in the characteristics of individuals primarily seeking treatment for alcohol and other drugs. It is also possible that the differences between groups may be more related to other differences (e.g. in socio-economic status, poly-substance use, health, or access to services) that correlate with the PDOCs, rather than to the PDOC itself.

There are a number of limitations to consider when reviewing these findings. Despite the use of validated scales, the study relied on participant self-report, with no objective measures of substance use or verification of service use. In addition, while the sample was large and purposively sampled from a diverse range of treatment services across two states, there was an over-sampling of residential clients, which we attempted to correct in the present analyses by applying weights based on treatment type. This was not a random sample, and selection bias may therefore have influenced our findings. Selection bias may also have arisen from our inability to assess all eligible participants: of 972 clients referred to the study who were not found to meet exclusion criteria, 18 % could not be contacted or declined to participate, and we do not know how many additional clients of these services were not referred by treatment staff for screening. It is possible that those who were referred, were contactable, and agreed to participate may represent a more stable end of the spectrum of problem severity. Comparison to Australian treatment data suggests that the sample was representative in terms of PDOC and age (aside from the exclusion of clients aged under 18 years), but may have contained less males and less Aboriginal or Torres Strait Islander participants than would have been expected in a random sample recruited from all Australian states and territories. As we could only assess the representativeness of the sample on these variables, there may have been other unmeasured variables that could have biased analyses and reduced generalisability to Australian AOD treatment-seekers. Nevertheless, the findings are consistent with previous studies examining clients in AOD treatment, with Patient Pathways being the largest treatment cohort study to compare alcohol to other drug treatment-seekers recruited from within the same service system across many variables. The inclusion of multiple measures relating to each type of social disadvantage corroborates findings within the study (e.g. differences in criminal justice problems and homelessness were, to some extent, reflected in differences in legal aid and housing service use).

## Conclusions

Individuals with alcohol as their PDOC comprise nearly half of AOD treatment clients and tend to experience less socio-economic marginalisation and poly-substance use than those with other drugs as their PDOC. However, their poor psychological health and elevated rates of ambulance and GP attendances may suggest greater health problems. Moreover, their older age, relative to those with other drugs as the PDOC, suggests a longer period of problematic use and accumulated health issues, which is likely to impact on treatment outcomes. The relative social acceptability of heavy drinking in Australia and pervasive availability of alcohol, relative to illicit drugs, may also make it more difficult for heavy drinkers to identify problems earlier, to seek treatment for such problems, or to maintain changes in drinking behaviour following treatment.

In summary, the present study provides further support for the complexity and severity of AOD treatment presentations. It also provides unique evidence within an Australian setting that clients with alcohol as their PDOC may differ in terms of their characteristics to those with other drugs as their PDOC. However, similarities across PDOC groups highlight that treatment still needs to be responsive to the broad range of issues faced by clients entering AOD treatment. Indeed, these findings reinforce previous calls [34, 35] regarding the need for integration or coordination of care between AOD treatment programs and services that address the broad range of complexities clients frequently present with (e.g. housing, employment, legal aid, medical, and mental health services, among others).
